# On exploring cross-sectional stability and persistence of microbiome in a multiple body site colorectal cancer dataset

**DOI:** 10.3389/fmicb.2025.1449642

**Published:** 2025-05-30

**Authors:** Hajra Ashraf, Sama Rezasoltani, Mohammad Mehdi Feizabadi, Seyedesomaye Jasemi, Hamid Asadzadeh Aghdaei, Zahra Bakudezfouli, Umer Zeeshan Ijaz, Leonardo A. Sechi

**Affiliations:** ^1^Department of Biomedical Sciences, University of Sassari, Sassari, Italy; ^2^Water and Environment Research Group, Mazumdar-Shaw Advanced Research Centre, University of Glasgow, Glasgow, United Kingdom; ^3^Division of Oral Microbiology and Immunology, Department of Operative Dentistry, Periodontology and Preventive Dentistry, RWTH Aachen University Hospital, Aachen, Germany; ^4^Department of Microbiology, School of Medicine, Tehran University of Medical Sciences, Tehran, Iran; ^5^Basic and Molecular Epidemiology of Gastrointestinal Disorders Research Center, Research Institute for Gastroenterology and Liver Diseases, Shahid Beheshti University of Medical Sciences, Tehran, Iran; ^6^National University of Ireland, Galway, Galway, Ireland; ^7^Department of Molecular and Clinical Cancer Medicine, University of Liverpool, Liverpool, United Kingdom; ^8^Complex Structure of Microbiology and Virology, AOU Sassari, Sassari, Italy

**Keywords:** colorectal cancer, 16S rRNA, microbiome, stability, multivariate statistics

## Abstract

There are several ways to recover signature microbiome of a disease pathology. One way is to look at the core microbiome, which comprises microbial species prevalent across majority of the samples. At a finer level, certain subcommunities may exhibit stable signature across the sampling space. There can also be similarity of differential patterns across different body sites. In view of above, and leveraging recent advancements in analytical strategies, we revisit a multi-factorial Iranian ColoRectal Cancer (CRC) dataset, and explore stable and persistent patterns in the microbiome. For this purpose, 16S rRNA gene is amplified from saliva and stool samples of CRC patients using healthy controls as a baseline (*n* = 80). The dataset is supplemented with demographical and nutritional data of the study participants that were collected through filled questionnaire. Our results indicate that certain microbial species i.e., *Actinobacteriota*, *Bifidobacterium*, *Prevotella* and *Fusobacterium* are consistently present in the CRC patients suggesting their potential as diagnostic biomarkers of disease. Additionally, we identified a group of microbes such as *Akkermansia*, *Selenomonas*, *Clostridia_UCG-014*, *Lautropia*, *Granulicatella*, *Bifidobacterium*, and *Gemella* that exhibit similar differential response across body sites irrespective of where they are found, whether in saliva or stool samples. This suggest that a part of saliva microbiome can act as a proxy for stool microbiome giving further credence to oral-gut axis. Overall, our findings underscore the importance of exploring stable microbial biomarkers in multifactorial CRC dataset by marginalizing out variabilities, with the potential for improved diagnosis and treatment strategies.

## Introduction

Colorectal cancer (CRC) is regarded as third most reported cancer around the world having a mortality rate of 1.8 million (Baidoun et al., 2021). The incidence of CRC is expected to rise globally, with an increase rate of 2.2 millions new cases and 1.1 millions deaths by 2030 particularly in western countries (Arnold et al., 2016). In Iran, CRC is prevalent in both genders, however, its ranked fourth most diagnosed cancer in males whilst second in Iranian females (Sung et al., 2021). It is believed that complex interplay between immune system, transcriptome, microbiome and metabolome might drive colon carcinogenesis and progression (Yang et al., 2019). The human gastrointestinal tract hosts a wide range of microbial community ∼10^13^ which surpass the number of genes in human genome by more than 150-fold. There is growing evidence implicating gut microbiota in the development of CRC via potential carcinogenic bacteria such as *Fusobacterium* as well as beneficial bacteria such as *Bifidobacterium* (Bullman et al., 2017; Wu et al., 2021; Yu et al., 2017). In our previous studies (Rezasoltani et al., 2022; Rezasoltani et al., 2024), we employed a comprehensive sampling strategy to understand microbial makeup in oral and fecal samples obtained from an Iranian CRC cohort. However, microbial ecology, particularly in the context of stable and persistent microbiome remains unexplored. Majority of the existing analytical strategies focus on divulging differential patterns from hypothesis-driven studies without considering the underpinning ecology that exerts pressure on the microbial communities either due to biotic or abiotic influences. Furthermore, the research studies are increasingly incorporating additional datasets and multi-omics modalities which leads to development of new class of methods designed to not only explore discriminating features (typically a subset) across study design (in a case-control, spatial or temporal setting), but also find correlations of subset of features across datasets (Ijaz et al., 2024). Therefore, guided by these recent developments, in this paper, we revisit the dataset, and:

a) apply a recently developed dynamic approach (Shade and Stopnisek, 2019) that recovers core microbiome by considering site-specific occupancies, and then fits a neutral model to further categorize the core microbiome into those that can be deterministically explained (either selected by host pressure or dispersal limitation) and those that are neutral. Note that the core microbial species typically exhibit functional redundancy, which stabilizes the ecosystem and possess specialized functions that can shape the microbiome landscape. The definition of core microbiome is debatable, and often implies a crisp “prevalence” threshold, i.e., how many samples should a microbe be observed in to tag it as part of core. These thresholds differ for different body sites and diseases (Shetty et al., 2017) and hence an incentive to use a more dynamic strategy;
b) recover ensembles of stable microbial subcommunities through a recently developed Ensemble Quotient Optimization algorithm (Shan et al., 2023), where collated relative abundance of these subcommunities either remain stable, or show a step-response. Note that whilst the overall proportion of the microbial subcommunities remain stable, the constituent members may adjust their abundances in relationship to each other; and
c) employ a multi-study group derivative of *sparse Projection to Latent Structure*—*Discriminant Algorithm* called MINT algorithm (Rohart et al., 2017) since we have a multi-factorial study design: group (healthy cohort [HC], CRC) X body site (saliva, stool). The aim is to recover microbial species that show a similar differential response between healthy individuals and CRC patients irrespective of which body site they are observed in.

The novelty in this study is to decipher those patterns which were not previously possible due to limitations of the traditional statistical approaches. Using the CRC dataset from Iranian study establishes two directions: to highlight disparities in saliva and stool microbiome specific to environmental exposures and dietary habits in Iran; and to go beyond the region-specific psychosocial stressors, marginalizing out variations, and establish a signature microbiome for CRC. The MINT algorithm, with its ability to recover inter-study nuances, should help in establishing biomarker signatures from saliva microbiome that are not only discriminant between healthy and CRC cohort but also act as a proxy for gut microbial profiles. These may potentially lead to developing diagnostic modalities.

## Materials and methods

### Bioinformatics

Our previous studies (Rezasoltani et al., 2022; Rezasoltani et al., 2024) provides an overview of the study design, sampling, and their processing. In brief, we collected saliva and stool samples of CRC patients and healthy controls. A total of 80 samples were screened for participation in this study in which only 78 underwent for final analysis due to low read numbers (< 5,000 reads).

Overall, we have obtained 13,571,662 paired end reads from 78 samples. On these, we recovered representative operational taxonomic units (OTUs) at 99% similarity using the same approach as used previously (Ijaz et al., 2018) with the modifications: (a) we have used the recent SILVA SSU Ref NR database release v.138 (Quast et al., 2012); and (b) we generated the rooted phylogenetic tree within the QIIME2 framework (Bolyen et al., 2019). Furthermore, we used PICRUSt2 (Douglas et al., 2020) within the QIIME environment to recover KEGG enzymes (10,543 enzymes for 78 samples) and MetaCyc pathway (489 enzymes for 78 samples) predictions for all the samples. For this purpose, we used the parameters –p-hsp-method pic –p-max-nsti 2 in qiime picrust2 full-pipeline.^1^ QIIME2 was also used to generate a final BIOM file that combined abundance information with the new taxonomy with a final *n* = 78 × *P* = 23,989 OTUs abundance table with the summary statistics of OTUs per sample as [Min: 49,786; 1st Quartile: 105,250; Median: 114,756; Mean: 113,357; 3rd Quartile: 123,510; Max: 159,166].

### Statistics

As a pre-processing step, we selected for samples with > 5,000 reads, removed typical contaminants such as Mitochondria and Chloroplasts, as well as any operational taxonomic units (OTUs) that were unassigned at all levels, as per recommendations given at https://docs.qiime2.org/2022.8/tutorials/filtering/, and then included only those samples relevant to this study, thus giving a final table of *n* = 78 × *P* = 23,370 OTUs with the summary statistics of reads mapping to these OTUs for samples as follows: [Min: 49,769; 1st Quartile: 104,419; Median: 113,840; Mean: 112,174;3rd Quartile: 122,755; Max: 159,159]. On this table, we have performed multivariate statistical analyses within in the context of meta data, with detailed provided in the Supplementary material.

## Results

### Core microbiota and neutral modeling

Significant differences (*p* < 0.05) are observed in richness estimates of OTUs between: HC saliva and CRC saliva; HC stool and CRC saliva; and HC saliva and CRC stool samples (Supplementary Figure 1). For MetaCyc pathways, we also observed significant differences (*p* < 0.05) in richness and Shannon entropy between HC saliva and CRC stool, as well as between HC stool and CRC saliva samples. Further exploration based on PERMANOVA suggested 3.7, 3, and 2.3% variation (*p* < 0.001) between HC and CRC in terms of composition, phylogeny, and metabolic function, respectively. PCoA analysis suggests distinct microbiota between stool and saliva samples giving credence to the presence of unique core microbiome.

**FIGURE 1:**
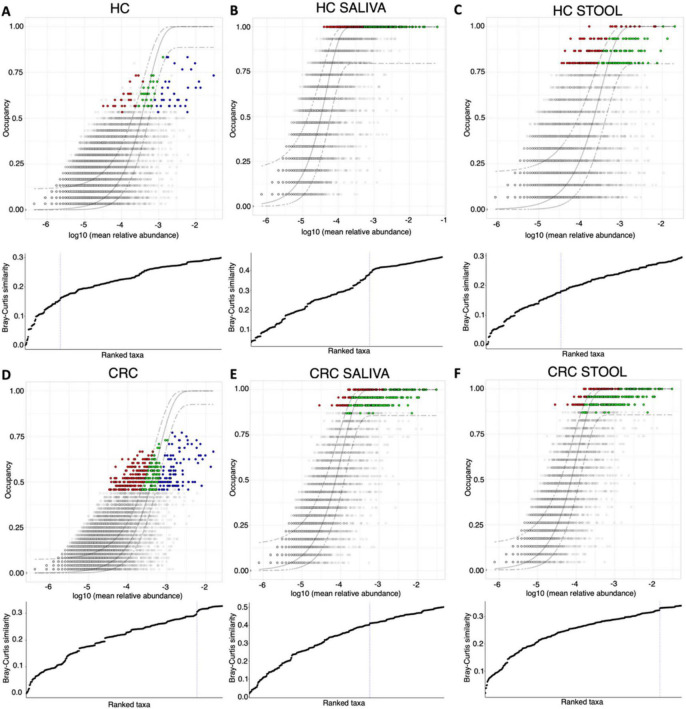
Core microbiome [red, green and blue points in **(A–F)**] identified through a dynamic approach and shown on species occupancy abundance diagrams. Six approaches are used: those that incorporate a site-specific occupancy criterion (occupancy being *saliva*, and *stool*) are shown in **(A)** HC and **(D)** CRC, respectively, whilst results with no site-specific occupancy are shown in **(B)** HC saliva, **(C)** HC stool, **(E)** CRC saliva, and **(F)** CRC stool, respectively. To identify the thresholds for core microbiome, we calculate the function C (that implicitly incorporates explanatory power of the chosen core subset in terms of capturing beta diversity) and is shown below the species occupancy diagram. The blue dotted line represents the threshold for “Last 2% decrease” criteria where OTUs are incorporated in the core subset until there is no more than 2% decrease in beta diversity. Independently, a neutral model is fitted with those OTUs that fall within the 95% interval confidence intervals shown in green, whilst non-neutral OTUs with observed frequency above the predicted frequency from the neutral model (selected by the host) are shown in red colors, and those with observed frequency below the predicted frequency from the neutral model (selected by dispersal limitation) are shown in green colors.

**FIGURE 2:**
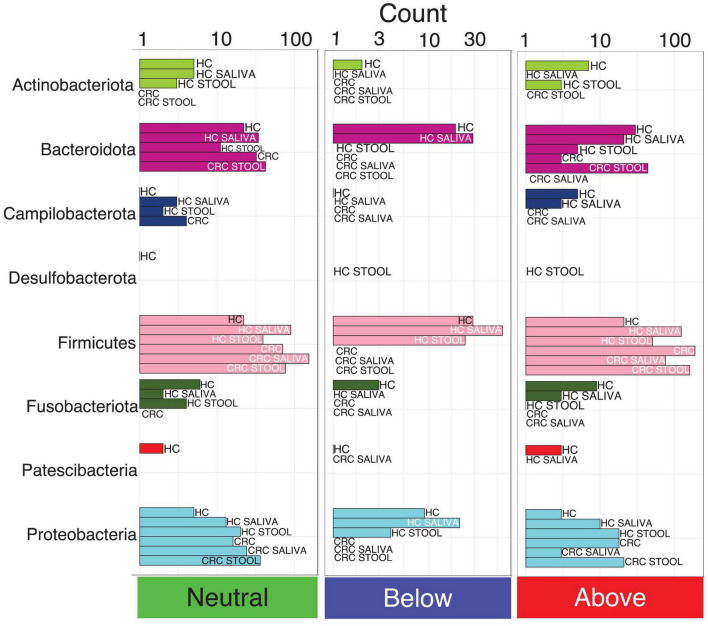
The count of core OTUs with taxonomic assignment at phylum level, and whether they are neutral, fitted below, or above the model, as per core microbiome inference in Figure 1. Phyla with fewer OTUs are not seen properly using a single scale, and are shown separately for each model in Supplementary Figure 3.

The core microbiome was then recovered using a dynamic approach where site-specific occupancy and replicate consistency within these sites decided which OTUs become part of the core microbiome (Figures 1, 2). Figures 1A, D show the results when site-specific occupancy (sites differing by body site) was used for HC, and CRC cohort, respectively. Figures 1B, C, E, F, show the results for HC saliva, HC stool, CRC saliva, and CRC stool, respectively, and employed no occupancy criteria. Using the occupancy criteria, the minimum prevalence threshold was observed to be ∼0.5 (Figure 1A) for the core OTUs in both HC and CRC cohort. Core OTUs detected in HC saliva (Figure 1B) were highly prevalent (∼1; Figure 1D). CRC Saliva (Figure 1E) on the other hand showed marked variation across cohorts with the minimum prevalence threshold of core OTUs detected at ∼0.86 and suggesting more inter-subject differences. The converse was observed for stool samples, where the detected minimum occupancy threshold was lower for HC (Figure 1C) than the CRC cohort (Figure 1F). The solid green points in occupancy-abundance diagrams are the core OTUs that fall within the 95% confidence interval of the fitted neutral model. The core OTUs that fall above (red) or below (blue) this range are considered deterministic, with particular interest in those that fall over the model as they are selected by the host pressure determined implicitly by the underlying environment.

Pie charts in Supplementary Figure 2 illustrates the proportional classification of core OTUs at phylum level. Across all the six models used in core microbiome analysis, majority of the core OTUs belonged to the phyla *Firmicutes*, *Campilobacterota* and *Proteobacteria*, whilst those belonging to *Patescibacteria*, *Campilobacterota* and *Fusobacteriota* were low. Notably, *Actinobacteriota* is the only phyla that distinguish HC saliva from CRC saliva albeit at a lower proportion of 1.61%. Core OTUs belonging to *Desulfobacterota* is exclusively observed in HC stool and is absent from CRC stool. Although the core microbiota is similar across all six models, their proportions differ. CRC saliva samples display more phyla level diversity in core OTUs as compared to the CRC stool samples. Neutrality analysis indicates that majority of the OTUs influenced by the host belonged to the phylum *Firmicutes*. There are more core OTUs belonging to *Firmicutes* and *Proteobacteria* selected by host (above the neutral model) for CRC cohort than for the healthy cohort (Figure 2). *Bacteroidota* on the other hand shows the opposite response, i.e., more core OTUs selected for HC than for CRC patients.

### Differential abundant taxa analysis taking into account paired nature of samples

Due to the paired nature of data, where multiple subjects provided both saliva and stool samples, respectively, we employed a specialized QCAT-C association test (Supplementary Figure 4). This test effectively minimizes Type 1 errors due to correlations introduced by paired nature and at the same times gives differential taxa at different lineages. Whilst on an average, the abundance profile showed a similar response between HC and CRC cohort for differential taxa on same body sites, there are some taxa that stood out. These include: *Halomonadaceae* family is low abundant in stool than in saliva for HC, with opposite response for CRC; *[Eubacterium]_brachy_group*, *Bifidobacterium*, and *Fusobacterium* genus significantly different between saliva and stool for HC than for CRC cohort; and *Actinobacteriota* phylum more abundant in CRC saliva than in HC saliva cohort.

### Stable microbial subcommunities identified through Ensemble Quotient Optimization (EQO) algorithm

We next identified microbial subcommunities (at genus level) where collated abundance of community members remained stable for each cohort (subcommunities for HC Saliva and CRC Saliva are shown in Supplementary Figure 5 whilst those of HC Stool and CRC Stool are shown in Supplementary Figure 6, respectively). The quality of fit is shown by Coefficient of Variation (CV) with lower values representing higher stability. Many of the genera were common in the stable microbial communities of saliva samples of HC and CRC cohort i.e., *Actinobacillus*, *Selenmonadaceae*, *Granulicatella*, *Selenomonas*, *Campylobacter*, *Saccharimonadaceae*, *Leptotrichia*, *Porphyromonas*, *Actinomyces*, *Neisseria*, *Fusobacterium*, *Veillonella*, *Prevotella* and *Streptococcus*. Those that were only part of stable cohort in CRC saliva are *Aggregatibacter*, *Bifidobacterium*, *Megasphaera*, *Gamella*, Stomatobaculum, *Atopobium*, *Rothia*, and *Lactobacillus* (Supplementary Figure 5). Similarly, those microbial genera that are common between CRC stool and HC stool are *Bilophila*, *Sutterella*, *Lachnospiraceae_NK4A136 group*, *Dialister*, *Muribaculaceae*, *Oscillospiraceae*; *UCG-10, Agathobacter*, *Oscillospiraceae*; *UCG-002, Alistipes*, *Christensenellaceae_R-7 group*, *Roseburia*, *Faecalibacterium*, *Escherichia-shigella*, *Barnesiellaceae*, *Prevotella* and, *Bacteroides*. Those genera that are part of stable cohort in CRC stool are *Eubacterium_siraeum* group, [*Ruminococcus*]*_torques_group*, *Dorea*, *Granulicatella*, *Lactobacillus*, *Pseudomonas*, *Paraprevotella*, *Bifidobacterium*, and *Streptococcus* (Supplementary Figure 6).

**FIGURE 3:**
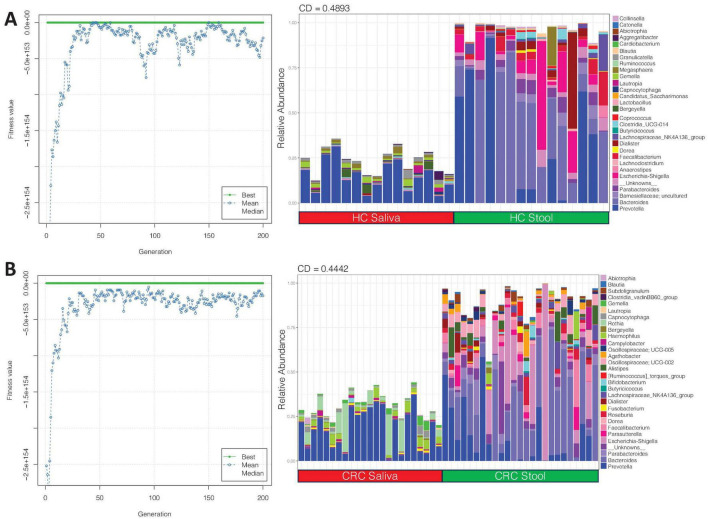
Shows the ensemble with differential response between saliva and stool samples for **(A)** HC and **(B)** CRC cohort. The left plots show the fitness value evolution of the genetic algorithm in finding these ensembles highlighting the convergence to a steady state solution whilst the right plots show the relative abundance profiles of these ensembles with coefficient of determination (CD) value, for which a larger absolute value implies stronger difference.

We then applied the EQO approach again, but this time, the aim is to find subcommunity whose relative abundance change across body sites, i.e., it had a differential response between stool and saliva samples for both HC, and CRC cohort (Figure 3). Membership of key genera in this subcommunity, with more proportional representation in stool (irrespective of whether the sample comes from HC or CRC) include: *Prevotella*, *Bacteroides*, *Parabacteroides*, *Escherichia-shigella*, and *Lachnospiraceae_NK4A136 group*. Some genera that were more abundant in HC saliva (in comparison to HC stool) include: *Prevotella*, *Gemella* and, *Megasphaera*, and those that have more proportional representation in CRC saliva (in comparison to CRC stool) include: *Prevotella* and, *Rothia*.

#### Clade level differential analyses using heat trees

Whilst previous differential analysis (QCAT-C) was done to show differences at different lineages (phylum, class, order, family, genus), we employed differential heat trees to see whether there are significant changes across lineages of the same node, or at clade level. The results are shown in Supplementary Figure 7 where the highlighted branches with different colors represent the significant enriched taxonomic paths in both groups (Brown: enriched in HC; Green: enriched in CRC). The tree highlights that *Fusobacteriota*, *Erysipelatoclostridiaceae* and *Lachnospiraceae* are the most discriminatory taxa between CRC and HCs cohorts as these are only dominant in CRC (Supplementary Figure 7).

#### Identifying inter-site biases using MINT analysis

**FIGURE 4:**
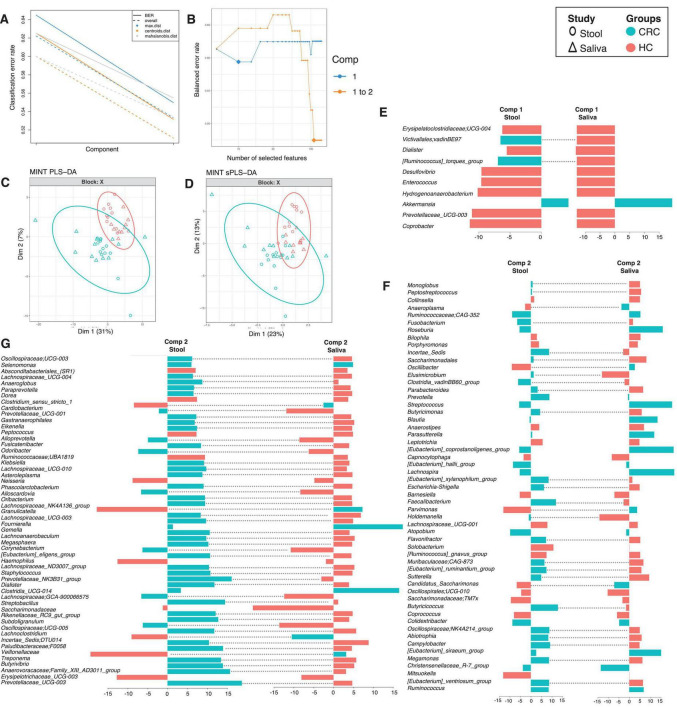
MINT results for inter-study comparison between HC and CRC for males, and across different body sites (stool and saliva). The algorithm is a two-step process where in **(A)**, two components were found that reduce the classification error rates (using centroids.dist in the function) in the algorithm, and in **(B)** shows the number of non-zero coefficients in these two components represented with a diamond. Panel **(C)** shows the reduced ordered representation of samples using all the genera in the first two components (MINT PLS-DA) with ellipse representing 95% confidence interval and percentage variations explained by these components in axes labels, whilst in **(D)** the same samples are shown but only using the discriminants from the two components (MINT sPLS-DA). Panels **(E–G)** then show the loading components for both studies, HC and CRC, with dotted lines connecting them if they disagree. The color of the bars in **(E–G)** show the category where the genera have maximum abundance across all groups considered. Note that in the loading diagrams, the length of the bar and not the directions of bar are important and highlights relative significance for that genus against others. Heatmap of these genera along with further information is displayed in Supplementary Figure 8.

**FIGURE 5:**
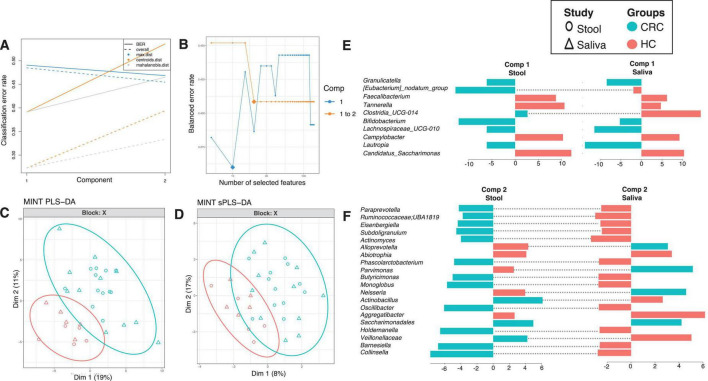
MINT results for inter-study comparison between HC and CRC for females, and across different body sites (stool and saliva). The algorithm is a two-step process where in **(A)**, two components were found that reduce the classification error rates (using centroids.dist in the function) in the algorithm, and in **(B)** shows the number of non-zero coefficients in these two components represented with a diamond. Panel **(C)** shows the reduced ordered representation of samples using all the genera in the first two components (MINT PLS-DA) with ellipse representing 95% confidence interval and percentage variations explained by these components in axes labels, whilst in **(D)** the same samples are shown but only using the discriminants from the two components (MINT sPLS-DA). Panels **(E,F)** then show the loading components for both studies, HC and CRC, with dotted lines connecting them if they disagree. The color of the bars in **(E,F)** show the category where the genera have maximum abundance across all groups considered. Note that in the loading diagrams, the length of the bar and not the directions of bar are important and highlights relative significance for that genus against others. Heatmap of these genera along with further information is displayed in Supplementary Figure 9.

To see which genera have similar differential response across body sites, we have employed a multi study group derivative of sparse Projection to Latent structure Discriminant Analysis (sPLS-DA) called MINT algorithm (also called P-Integration algorithm). The method gives non-zero loading vectors (weightage associated with differential taxa) for each study (Figures 4, 5) colored by the cohort these genera are upregulated in. Whilst several discriminatory genera were recovered, our focus lies solely on those, that show concordance across different body sites, whether saliva or stool. These are represented by the bars that are not joined by dotted lines in Figure 4 (for males) and Figure 5 (for females), and are the taxa that have a consistent response (same color), and thereby serve as diagnostic proxies. These include *Akkermansia*, *Selenomonas*, *Ruminococcaceae*; *CAG-352*, *Roseburia*, *Prevotella*, *Streptococcus*, *Blautia*, *Parasutterella*, *[Eubacterium]_coprostanoligenes_group*, *[Eubacterium]_hallii_group*, *Lachnospira*, *Atopobium*, *Colidextribacter*, *Christensenellaceae_R-7_group*, *Ruminococcus*, *Clostridia_UCG-014* and *Gemella* that are upregulated in CRC male cohort (as compared to HC), while *Granulicatella*, *Bifidobacterium*, *Lachnospiraceae_UCG-010*, *Lautropia*, and *Saccharimonadales* are upregulated in CRC female cohort (as compared to HC). *Erysipelatoclostridiaceae; UCG-004*, *Dialister*, *Desulfovibrio*, *Enterococcus*, *Prevotellaceae_UCG-003*, *Coprobacter*, *Hydrogenoanaerobacterium*, *Absconditabacteriales_(SR1)*, *Clostridium_sensu_stricto_1*, *Peptococcus*, *Klebsiella*, *Haemophilus*, *Erysipelotrichaceae_UCG-003*, *Collinsella*, *Bilophila*, *Porphyromonas*, *Anaerostipes*, *Leptotrichia*, *Capnocytophaga*, *Barnesiella*, *Lachnospiraceae_UCG-001*, *Solobacterium*, *[Ruminococcus]_gnavus_group*, *Oscillospirales; UCG-010*, *Saccharimonadaceae; TM7x*, *Coprococcus* and, *Mitsuokella* are upregulated in HC males whilst *Faecalibacterium*, *Tannerella*, *Campylobacter*, *Candidatus_Saccharimonas*, *Abiotrophia* and *Aggregatibacter* are upregulated in HC females.

Next, we wanted to explore if nutrition plays a differential role. For this purpose, we generated heatmaps of the discriminant genera identified from MINT algorithm for both males and females (Supplementary Figures 8, 9) and then applied hierarchical (using average linkages) clustering to identify clusters. By superimposing nutritional annotations, we did not find any clustering, neither for males nor for females. The samples mainly clustered together by Treatment (HC, and CRC), and Treatment2 (Stool and Saliva).

## Discussion

Growing literature highlights the role of microbiota in tumorigenesis and progression of CRC (Chen et al., 2020; Drewes et al., 2016; Mizutani et al., 2020; Wirbel et al., 2019). Despite this, the full understanding of the microbiome and its intricate interactions with the host remains incomplete and necessitates further investigation. The key to understanding how microbiota is linked to a particular disease is dependent on how stable the microbiota is and whether the patterns hold across majority of the subjects. In this study, we have revisited a CRC microbiome dataset (Rezasoltani et al., 2022; Rezasoltani et al., 2024) from the point of view of finding consistent, stable, and persistent microbiome. The analytical approaches used in this study offer several advantages. The dynamic core microbiome inference learns minimum occupancy threshold from the datasets, highlighting only those microbial taxa that remain consistently observed across all subjects. Based on this analysis, the dominant phyla are *Firmicutes*, *Campylobacterota* and *Proteobacteria* whilst *Patescibacteria*, *Campilobacterota* and *Fusobacteriota* are least abundant across all study cohorts. Notably, *Firmicutes* and *Proteobacteria* are selectively enriched in CRC Patients. *Firmicutes*, the most dominant phylum in the gut has mixed trends, with some species being beneficial while other species i.e., *Eubacterium eligens* and, *Eubacterium rectale* showing significant association with CRC (Sneath et al., 1986). In contrast, *Proteobacteria* are generally regarded as gut commensals with pathogenic features (Joly et al., 2010) that have a strong correlation with CRC (Sinha et al., 2016). A higher abundance of *Proteobacteria* in the gut is typically seen as gut dysbiosis indicator, suggesting a potential marker of diseases susceptibility (Shin et al., 2015; Xu et al., 2023).

We utilized the EQO algorithm to investigate stable microbial subcommunities within each cohort. These are subcommunities whose overall relative abundance remain stable whilst abundances of individual members of these subcommunities may vary in response to each other. In CRC saliva and stool samples, the membership of genera contributing to overall stability includes *Aggregatibacter*, *Megasphaera*, *Eubacterium_siraeum* group, *Dorea*, *Granulicatella*, *Paraprevotella*, *Bifidobacterium*, and *Streptococcus*. Notably, *Bifidobacterium* is the only genus present in both CRC saliva and stool, suggesting its potential as a prognostic marker. *Bifidobacterium* typically plays a role in intestinal epithelial cell differentiation and numerous studies have highlighted its beneficial effects, particularly in enhancing either the efficacy of immunotherapies or antitumor immunity which can suppress colon cancer (Ijaz et al., 2018; Rohart et al., 2017; Shan et al., 2023; Shetty et al., 2017). However, our studies observed elevated amount of *Bifidobacterium* both in the CRC saliva and stool supporting previous study conducted by Quast et al. (2012) who reported that 30% of the CRC patients have intratumor *Bifidobacterium*. This suggests *Bifidobacterium* might indicate mucosal barrier dysfunction or specific tumor characteristics.

Using EQO, we also identified subcommunities whose collated abundance (proportional abundance of member species) changes across body sites, showing differential responses of subcommunities between stool and saliva samples in both HC, and CRC cohorts. Genera more proportionally represented in the stool samples (regardless of whether the samples are from HC or CRC cohort) included *Prevotella*, *Bacteroides*, *Parabacteroides*, *Escherichia-shigella*, and *Lachnospiraceae_NK4A136* group. In contrast, genera that are more proportionally represented in CRC saliva compared to CRC stool included *Prevotella*, and *Rothia*. *Prevotella* species are gram negative anaerobes of the phylum *Bacteroidetes*, and are known to increase in cancer population due to elevated IL17 producing cells in the mucosa (Sobhani et al., 2011). Although generally considered commensals, some *Prevotella* species can act as opportunistic pathogens involved in endogenous infections (Brook, 2004). *Rothia*, a member of *Actinobacteria* phylum, has been associated with CRC (Rezasoltani et al., 2024). Typically, an oral commensal, *Rothia* is implicated in various diseases particularly in immunosuppressed hosts (Amer et al., 2017; Mougeot et al., 2020; Wang et al., 2021).

Our differential analyses have identified microbial species, strongly associated with CRC progression, notably *Fusobacteriota* and *Erysipelatoclostridiaceae*. *Fusobacterium nucleatum*, is often found at higher level in CRC patients, and is linked to inflammation and triggers immune responses that promote the production of inflammatory cells. It also induces immune suppression by modulating immune cells such as natural killer cells, T cells and macrophages (Hashemi Goradel et al., 2019; Wu et al., 2019). *Erysipelatoclostridiaceae*, an opportunistic bacterium associated with metabolic syndromes or other diseases (Nishino et al., 2018; Shao et al., 2017; Morgan et al., 2023) was found in higher abundance in CRC patients suggesting a potential link to CRC progression.

The use of MINT algorithm offers patterns that have a consistent response across multiple body sites, and do not change direction in terms of differential response between HC and CRC. If they are upregulated in HC as compared to CRC in stool, they exhibit the same response in saliva. The converse is also true, and has led to identification of *Akkermansia*, *Selenomonas*, and *Clostridia_UCG-014* as the key microbial genera in both stool and saliva of male CRC patients. *Akkermansia*, a gram-negative bacterium making up 1–4% of total human gut microbiota has been linked to various gastrointestinal diseases including Inflammatory bowel disease and cancer such as CRC (Gu et al., 2021; Wang et al., 2022). *Selenomonas*, and *Clostridium* also showed a close association with CRC patients supporting the previous published studies (Gao et al., 2017; Huo et al., 2022; Zwinsová et al., 2021). In contrast *Lautropia*, *Granulicatella*, and, *Bifidobacterium* were highly prevalent in female CRC patients validating existing literature (Costa et al., 2022; Kosumi et al., 2018; Li et al., 2020).

Overall, our research sheds light on the microbial ecology of individuals diagnosed with CRC, focusing on cross-sectional stability and persistence of the microbiome across multiple body sites, a largely unexplored area in scientific literature. We have utilized advanced statistical tools to unravel the complex interplay between microbiome, exposome, and other clinical parameters, whilst marginalizing for inconsistencies in results that may arise as a result of site-specific and subject-specific variabilities. Through a dynamic core microbiome approach that incorporates neutral modeling, we have further identified a signature microbiome associated with CRC, that is under selection pressure, as the microbial taxa that are fitted above the neutral model. These patterns demand exploration in further studies, not only for their diagnostic potential, but as target candidates for dietary intervention studies for treatment purposes. Furthermore, providing and analyzing stool samples may be inconvenient in a clinical setting. The MINT algorithm mitigates inter-study biases across body sites, enabling a direct gut-oral axis, and highlights the importance of using saliva swabs to give an account of microbes that are representative of CRC.

## Data Availability

The raw sequence files supporting the results of this article are available in the European Nucleotide Archive under the project accession number PRJEB76625 with details of the samples provided in Supplementary Data Sheet 1.

## Footnotes

1
https://github.com/picrust/picrust2/wiki/q2-picrust2-tutorial/

## Supplementary material

Supplementary Data Sheet 1 Metadata associated with the deposited sequences.

Supplementary Data Sheet 2 Source data for core microbiome analysis.

Supplementary Data Sheet 3 Supplementary material associated with this study.

## References

[B1] Amer A. Galvin S. Healy C. Moran G. (2017). The microbiome of potentially malignant oral leukoplakia exhibits enrichment for fusobacterium, leptotrichia, campylobacter, and rothia species. *Front. Microbiol.* 8:2391. 10.3389/fmicb.2017.02391 29250055 PMC5717034

[B2] Arnold M. Sierra M. Laversanne M. Soerjomataram I. Jemal A. Bray F. (2016). Global patterns and trends in colorectal cancer incidence and mortality. *Gut* 66 683–691. 10.1136/gutjnl-2015-310912 26818619

[B3] Baidoun F. Elshiwy K. Elkeraie Y. Merjaneh Z. Khoudari G. Sarmini M. (2021). Colorectal cancer epidemiology: Recent trends and impact on outcomes. *Curr. Drug Targets* 22 998–1009. 10.2174/1389450121999201117115717 33208072

[B4] Bolyen E. Rideout J. Dillon M. Bokulich N. Abnet C. Al-Ghalith G. (2019). Reproducible, interactive, scalable and extensible microbiome data science using QIIME 2. *Nat. Biotechnol.* 37 852–857. 10.1038/s41587-019-0209-9 31341288 PMC7015180

[B5] Brook I. (2004). Anaerobic pulmonary infections in children. *Pediatr. Emerg. Care* 20 636–640. 10.1097/01.pec.0000139751.63624.0b 15599270

[B6] Bullman S. Pedamallu C. Sicinska E. Clancy T. Zhang X. Cai D. (2017). Analysis of Fusobacterium persistence and antibiotic response in colorectal cancer. *Science* 358 1443–1448. 10.1126/science.aal5240 29170280 PMC5823247

[B7] Chen Y. Yang Y. Gu J. (2020). Clinical implications of the associations between intestinal microbiome and colorectal cancer progression. *Cancer Manag. Res.* 12 4117–4128. 10.2147/CMAR.S240108 32606919 PMC7295108

[B8] Costa C. Vieira P. Mendes-Rocha M. Pereira-Marques J. Ferreira R. Figueiredo C. (2022). The tissue-associated microbiota in colorectal cancer: A systematic review. *Cancers* 14:3385. 10.3390/cancers14143385 35884445 PMC9317273

[B9] Douglas G. Maffei V. Zaneveld J. Yurgel S. Brown J. Taylor C. (2020). PICRUSt2 for prediction of metagenome functions. *Nat. Biotechnol.* 38 685–688. 10.1038/s41587-020-0548-6 32483366 PMC7365738

[B10] Drewes J. Housseau F. Sears C. (2016). Sporadic colorectal cancer: Microbial contributors to disease prevention, development and therapy. *Br. J. Cancer* 115 273–280. 10.1038/bjc.2016.189 27380134 PMC4973155

[B11] Gao R. Kong C. Huang L. Li H. Qu X. Liu Z. (2017). Mucosa-associated microbiota signature in colorectal cancer. *Eur. J. Clin. Microbiol. Infect. Dis.* 36 2073–2083. 10.1007/s10096-017-3026-4 28600626

[B12] Gu Z. Pei W. Zhang Y. Zhu J. Li L. Zhang Z. (2021). Akkermansia muciniphila in inflammatory bowel disease and colorectal cancer. *Chin. Med. J.* 134 2841–2843. 10.1097/CM9.0000000000001829 34711719 PMC8667969

[B13] Hashemi Goradel N. Heidarzadeh S. Jahangiri S. Farhood B. Mortezaee K. Khanlarkhani N. (2019). *Fusobacterium nucleatum* and colorectal cancer: A mechanistic overview. *J. Cell. Physiol.* 234 2337–2344. 10.1002/jcp.27250 30191984

[B14] Huo R. Wang Y. Hou S. Wang W. Zhang C. Wan X. (2022). Gut mucosal microbiota profiles linked to colorectal cancer recurrence. *World J. Gastroenterol.* 28 1946–1964. 10.3748/wjg.v28.i18.1946 35664963 PMC9150055

[B15] Ijaz U. Ameer A. Saleem F. Gul F. Keating C. Javed S. (2024). Specialty grand challenge: How can we use integrative approaches to understand microbial community dynamics? *Front. Media SA* 4:1432791. 10.3389/fsysb.2024.1432791 PMC12342006 40809114

[B16] Ijaz U. Sivaloganathan L. McKenna A. Richmond A. Kelly C. Linton M. (2018). Comprehensive longitudinal microbiome analysis of the chicken cecum reveals a shift from competitive to environmental drivers and a window of opportunity for campylobacter. *Front. Microbiol.* 9:2452. 10.3389/fmicb.2018.02452 30374341 PMC6196313

[B17] Joly F. Mayeur C. Bruneau A. Noordine M. Meylheuc T. Langella P. (2010). Drastic changes in fecal and mucosa-associated microbiota in adult patients with short bowel syndrome. *Biochimie* 92 753–761. 10.1016/j.biochi.2010.02.015 20172013

[B18] Kosumi K. Hamada T. Koh H. Borowsky J. Bullman S. Twombly T. (2018). The amount of bifidobacterium genus in colorectal carcinoma tissue in relation to tumor characteristics and clinical outcome. *Am. J. Pathol.* 188 2839–2852. 10.1016/j.ajpath.2018.08.015 30243655 PMC6284552

[B19] Li D. Xi W. Zhang Z. Ren L. Deng C. Chen J. (2020). Oral microbial community analysis of the patients in the progression of liver cancer. *Microb. Pathog.* 149:104479. 10.1016/j.micpath.2020.104479 32920149

[B20] Mizutani S. Yamada T. Yachida S. (2020). Significance of the gut microbiome in multistep colorectal carcinogenesis. *Cancer Sci.* 111 766–773. 10.1111/cas.14298 31910311 PMC7060472

[B21] Morgan P. A. Parbie P. K. Ntiamoah D. O. Boadu A. A. Asare P. Lamptey I. N. K. (2023). Gut microbiome variation in pulmonary TB patients with diabetes or HIV comorbidities. *Front. Microbiomes* 2:1123064. 10.3389/frmbi.2023.1123064 PMC12993506 41853362

[B22] Mougeot J. Beckman M. Langdon H. Brennan M. Bahrani Mougeot F. (2020). Oral microbiome signatures in hematological cancers reveal predominance of actinomyces and rothia species. *J. Clin. Med.* 9:4068. 10.3390/jcm9124068 33348567 PMC7767039

[B23] Nishino K. Nishida A. Inoue R. Kawada Y. Ohno M. Sakai S. (2018). Analysis of endoscopic brush samples identified mucosa-associated dysbiosis in inflammatory bowel disease. *J. Gastroenterol.* 53 95–106. 10.1007/s00535-017-1384-4 28852861

[B24] Quast C. Pruesse E. Yilmaz P. Gerken J. Schweer T. Yarza P. (2012). The SILVA ribosomal RNA gene database project: Improved data processing and web-based tools. *Nucleic Acids Res.* 41 D590–D596. 10.1093/nar/gks1219 23193283 PMC3531112

[B25] Rezasoltani S. Aghdaei H. Jasemi S. Gazouli M. Dovrolis N. Sadeghi A. (2022). Oral microbiota as novel biomarkers for colorectal cancer screening. *Cancers* 15:192. 10.3390/cancers15010192 36612188 PMC9818409

[B26] Rezasoltani S. Azizmohammad Looha M. Asadzadeh Aghdaei H. Jasemi S. Sechi L. Gazouli M. (2024). 16S rRNA sequencing analysis of the oral and fecal microbiota in colorectal cancer positives versus colorectal cancer negatives in Iranian population. *Gut Pathog.* 16 1–13. 10.1186/s13099-024-00604-0 38378690 PMC10880352

[B27] Rohart F. Eslami A. Matigian N. Bougeard S. Lê Cao K. A. (2017). MINT: A multivariate integrative method to identify reproducible molecular signatures across independent experiments and platforms. *BMC Bioinformatics* 18:128. 10.1186/s12859-017-1553-8 28241739 PMC5327533

[B28] Shade A. Stopnisek N. (2019). Abundance-occupancy distributions to prioritize plant core microbiome membership. *Curr. Opin. Microbiol.* 49 50–58. 10.1016/j.mib.2019.09.008 31715441

[B29] Shan X. Goyal A. Gregor R. Cordero O. (2023). Annotation-free discovery of functional groups in microbial communities. *Nat. Ecol. Evol.* 7 716–724. 10.1038/s41559-023-02021-z 36997739

[B30] Shao T. Shao L. Li H. Xie Z. He Z. Wen C. (2017). Combined signature of the fecal microbiome and metabolome in patients with gout. *Front. Microbiol.* 8:268. 10.3389/fmicb.2017.00268 28270806 PMC5318445

[B31] Shetty S. Hugenholtz F. Lahti L. Smidt H. de Vos W. (2017). Intestinal microbiome landscaping: Insight in community assemblage and implications for microbial modulation strategies. *FEMS Microbiol. Rev.* 41 182–199. 10.1093/femsre/fuw045 28364729 PMC5399919

[B32] Shin N. Whon T. Bae J. (2015). *Proteobacteria*: Microbial signature of dysbiosis in gut microbiota. *Trends Biotechnol.* 33 496–503. 10.1016/j.tibtech.2015.06.011 26210164

[B33] Sinha R. Ahn J. Sampson J. Shi J. Yu G. Xiong X. (2016). Fecal microbiota, fecal metabolome, and colorectal cancer interrelations. *PLoS One* 11:e0152126. 10.1371/journal.pone.0152126 27015276 PMC4807824

[B34] Sneath P. Mair N. Sharpe M. Holt J. (1986). *Bergey’s Manual of Systematic Bacteriology*, Volume 2. Philadelphia, PA: Williams & Wilkins.

[B35] Sobhani I. Tap J. Roudot-Thoraval F. Roperch J. Letulle S. Langella P. (2011). Microbial dysbiosis in colorectal cancer (CRC) patients. *PLoS One* 6:e16393. 10.1371/journal.pone.0016393 21297998 PMC3029306

[B36] Sung H. Ferlay J. Siegel R. Laversanne M. Soerjomataram I. Jemal A. (2021). Global cancer statistics 2020: Globocan estimates of incidence and mortality worldwide for 36 cancers in 185 countries. *CA Cancer J. Clin.* 71 209–249. 10.3322/caac.21660 33538338

[B37] Wang F. Cai K. Xiao Q. He L. Xie L. Liu Z. (2022). Akkermansia muciniphila administration exacerbated the development of colitis-associated colorectal cancer in mice. *J. Cancer* 13 124–133. 10.7150/jca.63578 34976176 PMC8692691

[B38] Wang Y. Zhang Y. Qian Y. Xie Y. Jiang S. Kang Z. (2021). Alterations in the oral and gut microbiome of colorectal cancer patients and association with host clinical factors. *Int. J. Cancer* 10.1002/ijc.33596 Online ahead of print. 33844851

[B39] Wirbel J. Pyl P. Kartal E. Zych K. Kashani A. Milanese A. (2019). Meta-analysis of fecal metagenomes reveals global microbial signatures that are specific for colorectal cancer. *Nat. Med.* 25 679–689. 10.1038/s41591-019-0406-6 30936547 PMC7984229

[B40] Wu J. Li Q. Fu X. (2019). *Fusobacterium nucleatum* contributes to the carcinogenesis of colorectal cancer by inducing inflammation and suppressing host immunity. *Transl. Oncol.* 12 846–851. 10.1016/j.tranon.2019.03.003 30986689 PMC6462820

[B41] Wu Y. Jiao N. Zhu R. Zhang Y. Wu D. Wang A. (2021). Identification of microbial markers across populations in early detection of colorectal cancer. *Nat. Commun.* 12:3063. 10.1038/s41467-021-23265-y 34031391 PMC8144394

[B42] Xu Y. Zhao J. Ma Y. Liu J. Cui Y. Yuan Y. (2023). The microbiome types of colorectal tissue are potentially associated with the prognosis of patients with colorectal cancer. *Front. Microbiol.* 14:1100873. 10.3389/fmicb.2023.1100873 37025624 PMC10072283

[B43] Yang Y. Misra B. Liang L. Bi D. Weng W. Wu W. (2019). Integrated microbiome and metabolome analysis reveals a novel interplay between commensal bacteria and metabolites in colorectal cancer. *Theranostics* 9 4101–4114. 10.7150/thno.35186 31281534 PMC6592169

[B44] Yu T. Guo F. Yu Y. Sun T. Ma D. Han J. (2017). *Fusobacterium nucleatum* promotes chemoresistance to colorectal cancer by modulating autophagy. *Cell* 170 548–563.e16. 10.1016/j.cell.2017.07.008 28753429 PMC5767127

[B45] Zwinsová B. Petrov V. Hrivòáková M. Smatana S. Micenková L. Kazdová N. (2021). Colorectal tumour mucosa microbiome is enriched in oral pathogens and defines three subtypes that correlate with markers of tumour progression. *Cancers* 13:4799. 10.3390/cancers13194799 34638284 PMC8507728

