# Supplementary material for: On exploring cross-sectional stability and persistence of microbiome in a multiple body site colorectal cancer dataset
Source: Front Microbiol. 2025 May 30;16:1449642. doi: 10.3389/fmicb.2025.1449642 (PMC12162472; doi:10.3389/fmicb.2025.1449642)
Supplement: Supplementary Data Sheet 3 — Supplementary material associated with this study. [file Data_Sheet_3.pdf]

Supplementary Materials

On exploring cross-sectional stability and persistence of microbiome in a multiple body site colorectal cancer dataset

Hajra Ashraf[1,2], Sama Rezasoltani[3], Mohammad Mehdi Feizabadi[4], Seyedesomayeh Jasemi[1], Hamid Asadzadeh Aghdaei[5], Zahra Bakudezfouli[5], Umer Zeeshan Ijaz[2,6,7, *], Leonardo A. Sechi[1,8, *]

[1]Department of Biomedical Sciences; University of Sassari, Sassari, Italy
[2]Water & Environment Research Group, University of Glasgow, Mazumdar-Shaw Advanced Research Centre, Glasgow, United Kingdom
[3]Division of Oral Microbiology and Immunology, Department of Operative Dentistry, Periodontology and Preventive Dentistry, RWTH University Hospital, 52057 Aachen, Germany
[4]Department of Microbiology, School of Medicine, Tehran University of Medical Sciences, Tehran 19835-178, Iran
[5]Basic and Molecular Epidemiology of Gastrointestinal Disorders Research Center, Research Institute for Gastroenterology and Liver Diseases, Shahid Beheshti University of Medical Sciences, Tehran 19835-178, Iran.
[6]National University of Ireland, Galway, University Road, Galway, Ireland
[7]Department of Molecular and Clinical Cancer Medicine, University of Liverpool, Liverpool, United Kingdom
[8]Complex Structure of Microbiology and Virology; AOU Sassari, Sassari, Italy

* **Joint corresponding authors** (both authors jointly directed this work)

Contact:
Hajra Ashraf, h.ashraf@studenti.uniss.it
Umer Zeeshan Ijaz*, Umer.Ijaz@glasgow.ac.uk
(http://userweb.eng.gla.ac.uk/umer.ijaz)
Leonardo A. Sechi*, sechila@uniss.it

Materials and Methods

The R's vegan package [1] was used for alpha and beta diversity analyses. For alpha diversity measures we have used (after rarefying to minimum library size): (i) *Shannon entropy* – a commonly used index to measure balance within a community; (ii) *Chao2 richness* – the estimated number of species/features in an incidence table. We have used R's aov() function to calculate the pair-wise analysis of variance (ANOVA) p-values which were then drawn on top of alpha diversity figures. To adjust for subject IDs, since a single patient provided both saliva and gut sample, all of the pairwise statistics were done using one within subject factor in anova, as aov(value ~ Groups + Error(SubjectID/Groups)).
To visualise the abundance table, we have used Principal Coordinate Analysis (PCoA) with different distance measures. Specifically, we have used three different measures in PCoA: (i) *Bray-Curtis distance* on the OTU abundance table to visualise the compositional changes; (ii) *Unweighted UniFrac distance* estimated using R's Phyloseq package [2] to see changes between samples in terms of phylogeny; and (iii) *Hierarchical Meta-Storms* (HMS) [3], a recent functional beta diversity distance which takes the observed KEGG Orthologs (KOs) recovered from the dataset, and then calculates the functional beta diversity distance in a hierarchical fashion propagating the KOs abundances upward to the pathways in a multi-level pathway hierarchy to give a weighted dissimilarity measure. Additionally, Vegan package was also used to perform PERMANOVA analyses to see if the microbial or functional community structures can be explained by different sources of variability.

We have used DESeqDataSetFromMatrix() function from DESeq2 package [4] with the adjusted *p*-value significance cut-off of 0.05 and log2 fold change cut-off of 2. This function uses negative binomial GLM to obtain maximum likelihood estimates for log fold change of

KEGG KOs between any two conditions considered in this study. Afterwards, Bayesian shrinkage is applied to obtain shrunken log fold changes subsequently employing the Wald test for obtaining significances. To visualise the differentially abundant KOs we have used *Microbial Metabolism* widget on iPATH3 https://pathways.embl.de/

To identify core microbiome, we have used the approach discussed in [5], and further explained in Figure 9 of [6]. In scientific literature, there is no real consensus at what thresholds of occupancy (alternatively, referred to as prevalence, i.e., what proportion of the total number of samples a microbe is observed in) constitutes the core microbiome membership. This is further convoluted by inter-subject variabilities across body-sites. For example, whilst gut communities remain taxonomically, and functionally stable, some other body sites, e.g., vaginal microbial communities are not very robust [7]. Therefore, the membership threshold in terms of occupancy typically varies from 30% to 85%. To circumvent this problem, the dynamic approach of [5] first ranks the OTUs by occupancy, and then calculates the minimal occupancy threshold dynamically by learning from the data. The ranking of OTUs is done using a combination of two metrics: *site-specific occupancy* (whether samples are grouped by different treatment groups); and replicate consistency (whether the OTUs are consistent across replicates in the above treatment group). We have used 6 models: in two models we have used *site-specific occupancy* for HC and CRC (sites being Stool and Saliva); whilst for remaining four models (HC_Saliva, HC_Stool, CRC_Saliva, and CRC_Stool), we have not used any site-specific occupancy and instead clumped all the samples together under a single category. After ranking the OTUs, the subset of core taxa is constructed incrementally by adding one OTU at a time to the core set of OTUs, from highly prevalent to lowly prevalent ones. The contribution of the core subset to beta diversity is then calculated every time a new OTU becomes member of the core set using the Bray-Curtis distance in the equation, $C = 1 - \frac{BC_{core}}{BC_{all}}$. The original authors have specified a threshold at which the core subset construction stops, i.e., where the addition of an OTU does not cause more than 2% increase in the explanatory value by Bray-Curtis distance. Independently, a neutral model [8] is fitted to the "S" shaped abundance-occupancy distributions inform the OTUs that are likely selected by the environment. These are obtained as those that fall outside the 95% confidence interval of the fitted model, and are inferred to be deterministically assembled, rather than neutrally selected, with those that are **above the model selected by the host environment** (represented by red colour), and those points below the model **are dispersal limited** (represented by blue colour). The taxonomy tree of the core microbiome across different breeds and treatment groups were drawn using the R's metacoder package [9].

To identify taxa that were changing significantly in abundance between different groups on the taxonomic tree, we used the differential heat trees approach package [9] which highlights differential features on the recovered taxonomy. Briefly the method constructs a taxonomic tree after removing low occurring OTUs (i.e., those that have all counts with less than 5 reads). Afterwards, each OTU is represented as a proportional representation in a sample. For each taxon, a Wilcoxon Rank Sum test is used to test for differences between the median abundances of samples in each treatment, with the p-values saved. These p-values are then adjusted for multiple comparison using method="fdr" in p.adjust() function. The resulting nodes, if significant in the taxonomic tree, are then colored according to log2 ratio of median proportions.

Since we have a nested design, i.e., microbial community data from HC and CRC samples for both Saliva and Stool groups, we have used the Multivariate Integration (MINT) algorithm [10] to enable inter study comparison between HC and CRC cohort, and further explained in Figure 10 of [6]. The algorithm is an extension of the multi-group Projection to Latent Structure (mgPLS), and it attempts to find a common projection space across all studies (three categories as mentioned-above), defined on a small subset of discriminative variables that

consistently discriminate the outcome classes (Saliva and Stool). In MINT, we have combined $M = 2$ datasets denoted $X^{(1)}(N_1 \times P)$, and $X^{(2)}(N_2 \times P)$, where both datasets share the P genera whilst the number of samples differ, i.e., N_1, N_2,..., N_3. All studies have associated dummy indicator outcome $Y^{(1)}$, $Y^{(2)}$,..., $Y^{(3)}$ in which the status of samples whether it is stool or saliva is coded. MINT then solves the problem: $\max_{a_h, b_h} \sum_{m=1}^{M} N_m \text{cov}(X_h^{(m)} a_h, Y_h^{(m)} b_h)$, with the constraints$\|a_h\|_2 = 1$ and $\|a_h\|_1 \leq \lambda$, where the covariance of scores between the datasets are maximised by finding the global loading vectors a_h and b_h common to all studies (akin to PCA analysis). The first constraint ensures the loading vector to have unit magnitude (requirement of the procedure) and the second constraint (also called l_1 penalty) to ensure that for the features that do not vary between the categories, the corresponding loading vector coefficients go to zero. This is done by using the sparsity control parameter λ in the above equation, and by adjusting it enforces shrinkage of loading vector coefficients. According to the recommendations given in mixOmics package (http://www.mixomics.org), before applying the procedure splsda(), we pre-filter 1% of the lowest abundant genera and then perform TSS+CLR (Total Sum Scaling followed by Centralised Log Ratio) normalisation. To predict the number of latent components (associated loading vectors) and the number of discriminants, the perf.plsda() and tune.splsda() functions were used, respectively. In the latter case, we fine tune the model was applied using leave-one-out cross-validation by splitting the data into training and testing sets and then finding the classification error rates employing overall error rates, between the predicted latent variables with the centroid of the class labels (categories considered in this study) using the centroid distance. The MINT algorithm is run separately for males and female due to gender differences in microbial ecology of healthy gut microbiomes [11].

Since the data for subjects are paired, i.e., same individuals have provided Saliva and Stool samples, and there exist strong correlations between them leading to type 1 errors, we have also used a specialised cluster association test [12] utilising R's miLineage package (https://tangzheng1.github.io/tanglab/software.html) with this test referred to as QCAT-C test using the QCAT_GEE. Cluster () function (with default values) from the package. The test is robust to deduce complex correlations that exist among microbes due to paired nature of samples. Additionally, the QCAT-C test is a two-part test where it fits separate models to microbes that are excessively zero, and those that are not, referred to as positive microbes, based on the taxonomic tree to localize the covariate-associated lineages. As a result, the differential abundance analysis of microbes gives better estimates and reduces Type 1 errors. To visualise the differentially abundant taxa at different taxonomic ranks, we have used *Total Sum Scaling* followed by a *Centralized Log Ratio* (TSS+CLR) transformation on the raw abundance values.

We next wanted to see if we can find a minimal subset of species that either remain stable or have a step-response with respect to the status of the sample, whether it is coming from HC or CRC. For this purpose, we have incorporated the Ensemble Quotient Optimisation (EQO) approach of Shan et al. 2023 [13], and further explained in Figure 6 of [6]. The approach is also different as compared to conventional approaches where the emphasis is on looking at differential patterns (across different sampling cohorts) of a single microbial species in isolation (although they are corrected for multiple testing). Instead, the goal here is to recover a group of species, called, "Ensemble", that may play a certain ecological role. The method uses a relative abundance table, called community matrix $\boldsymbol{M}$ (*P* taxa over *n* samples), where the goal is to obtain a vector $\boldsymbol{x} \in (0,1)^P$ where the ith position in the vector is either 0 or 1, i.e., a subset of species with values 1 belong to an ensemble which we are interested in recovering. This ensemble is recovered in the context of a phenotype/predictor variable $\boldsymbol{y}$ by optimizing an *Ensemble Quotient* $EQ = \frac{\boldsymbol{x}^T \boldsymbol{Q} \boldsymbol{x}}{\boldsymbol{x}^T \boldsymbol{P} \boldsymbol{x}}$, through a genetic algorithm, where $\boldsymbol{P}$ and $\boldsymbol{Q}$ are algebraic transformations of the community matrix that captures the covariance between taxa, and the covariance between taxa and $\boldsymbol{y}$. We have used $\boldsymbol{y}$ in two different ways: a) where we are

interested in the stable microbiome for both HC and CRC samples by considering $\boldsymbol{y}$ as a uniform variable i.e., consisting of 1s, with $\boldsymbol{Q} = \boldsymbol{M}^T\mathbf{1}\mathbf{1}^T\boldsymbol{M}$, and $\boldsymbol{P} = \boldsymbol{M}^T\boldsymbol{M} - \frac{2}{n}\boldsymbol{M}^T\mathbf{1}\mathbf{1}^T\boldsymbol{M} + \frac{1}{n^2}\boldsymbol{M}^T\mathbf{1}\mathbf{1}^T\mathbf{1}\mathbf{1}^T\boldsymbol{M}$; and b) where we are interested in stable microbiota that has a step response between Saliva and Stool samples for both HC and CRC samples, by using $\boldsymbol{Q} = \boldsymbol{M}_0^T\boldsymbol{Y}\boldsymbol{L}\boldsymbol{L}^T\boldsymbol{Y}^T\boldsymbol{M}_0$, and $\boldsymbol{P} = \boldsymbol{M}_0^T\boldsymbol{M}_0$ ($\boldsymbol{Y}$ is an augmented categorical matrix with n rows and containing 0 [Saliva] or 1 [Stool] depending on where the samples come from). For case a) we get *Coefficient of Variation* (CV), whilst for case b) *Coefficient of Determination* (CD) with a value approaching a high value representing a good fit. To optimize the EQ to obtain $\boldsymbol{x}$, we followed the genetic algorithm optimization located at https://github.com/Xiaoyu2425/Ensemble-Quotient-Optimization. In the genetic algorithm, we have used the following parameterizations: a population size of 100 solutions, maximum of 200 generations, and maximum 30 taxa to be returned as an ensemble. Additionally, we collated the OTUs at genus level, and retained only those taxa that occupied atleast 50% of the samples.

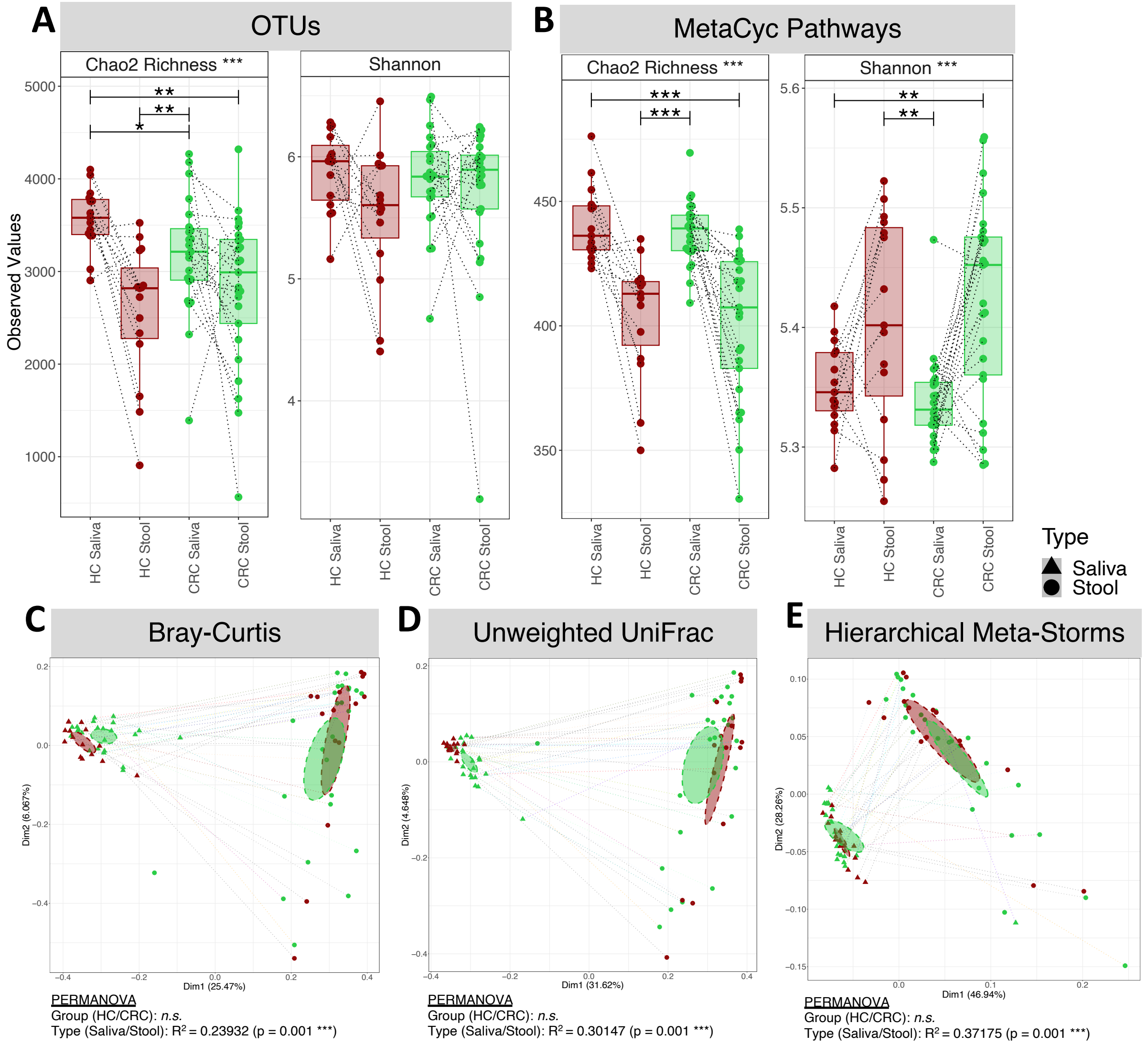

Figure S1: Alpha diversity (Chao 2 Richness and *Shannon* entropy) comparison of (A) bacterial OTUs, and (B) MetaCyc pathways predicted from the PICRUSt2 software. Beta diversity (C-E) represented by principal coordinate analysis (PCoA) plots with each axis showing the percentage variability explained by that axis, and where ellipses represent 95% confidence interval of the standard error for a given group. We have used three different distance measures: (C) *Bray-Curtis distance* to show differences in composition, (D) *Unweighted UniFrac distance* to show differences in phylogeny, and (E) *Hierarchical Meta-*

storms to show differences in metabolic function. PERMANOVA statistics utilising these distance measures are shown underneath the PCoA plots to suggest if there are significant differences between the groups with R^2 value showing percentage variability explained. The solid lines in panels with boxplots connect groups if one within subject ANOVA is significant with significance values as: * $p < 0.05$, ** $p < 0.01$, or *** $p < 0.001$. The dotted lines connect subjects that have provided both Saliva and Stool samples.

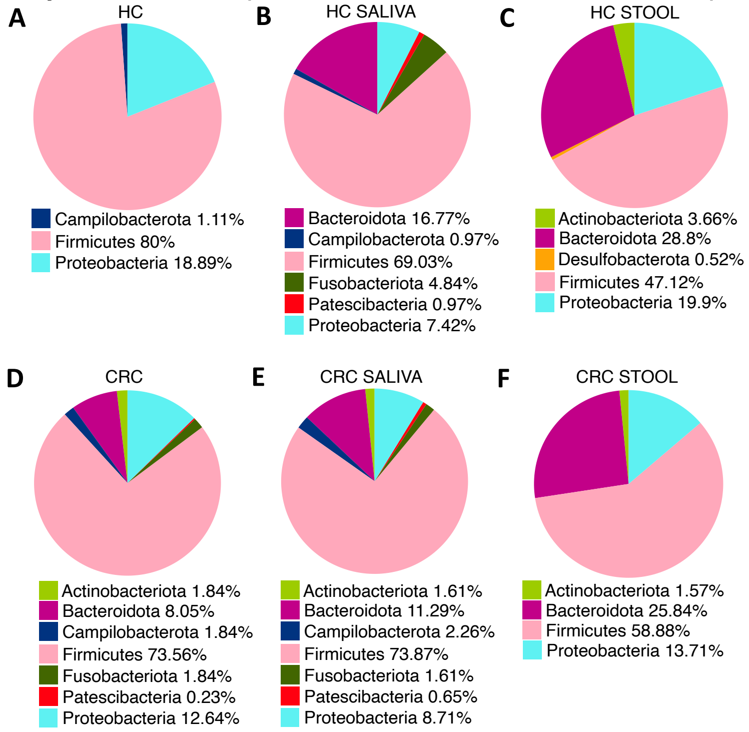

Figure S2: The proportion of core OTUs belonging to different phyla are shown with pie charts in (A) HC [occupancy: Saliva and Stool)], (B) HC Saliva, (C) HC Stool, (D) CRC [occupancy: Saliva and Stool], (E) CRC Saliva, and (F) CRC Stool, and correspond to the core microbiome estimated in Figure 2.

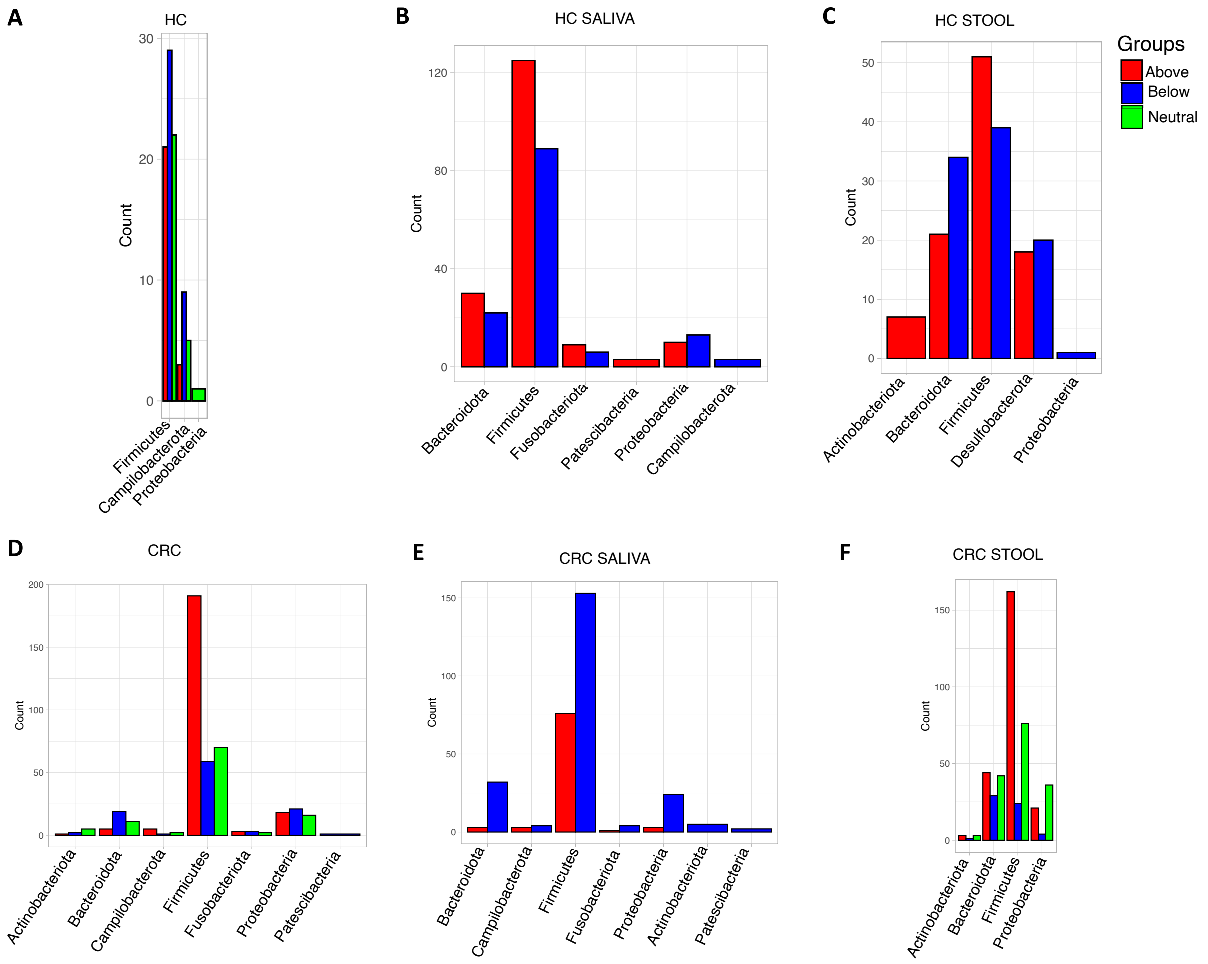

Figure S3. Neutral modelling results shown separately for each model from Figure 2 with the source data given in Supplementary_Data_Table_2.csv.

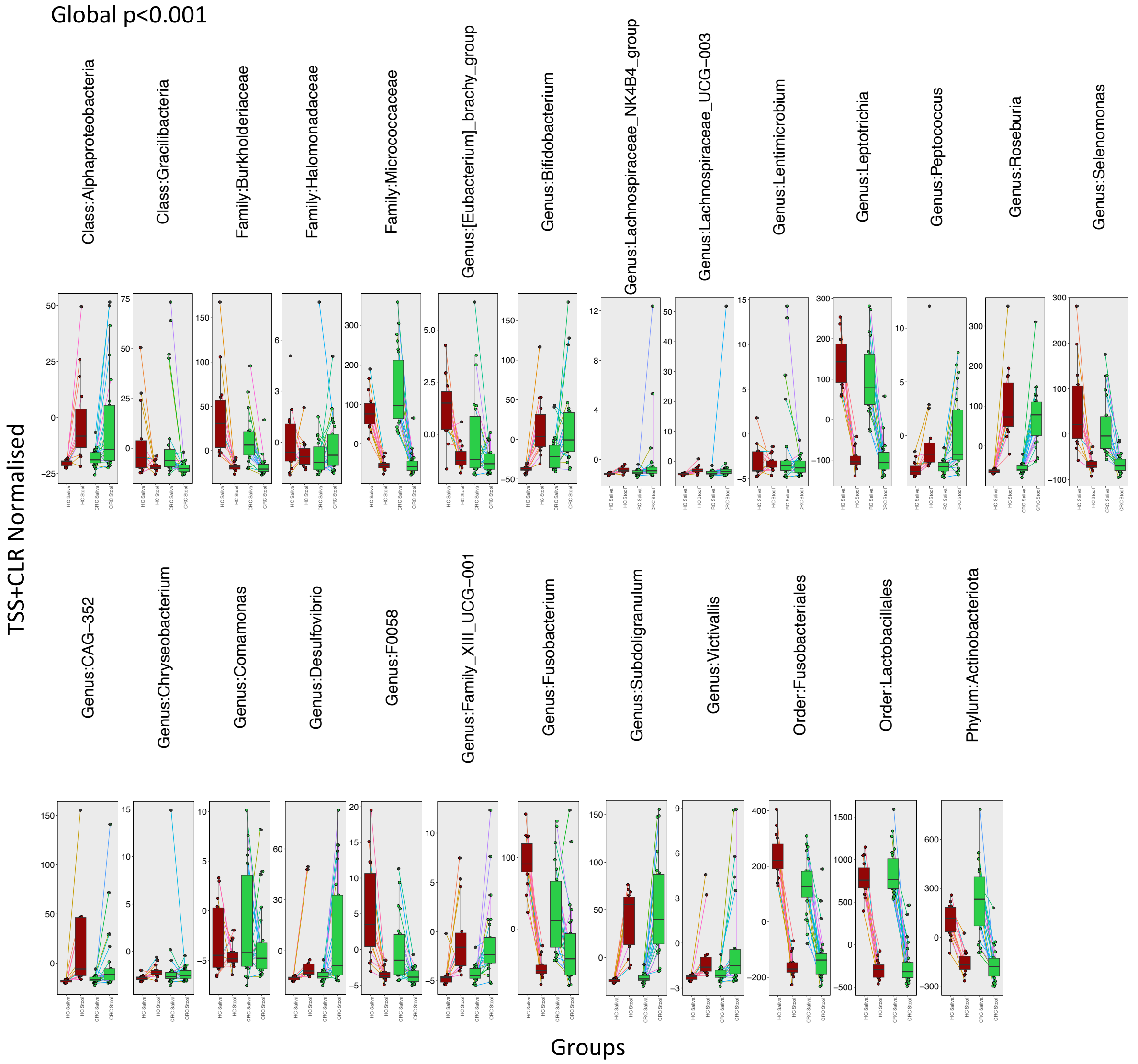

Figure S4: Subset of taxa (at different lineages, *Kingdom, Phylum, Class, Order, Family, Genus*) returned from QCAT-C association test that are differentially abundant between the cohorts considered in this study. The QCAT-C association test that takes into account paired nature of samples i.e., originating from the same subject, and are connected by lines. The values represent the TSS+CLR normalized abundances of individual taxa. The global P-value is the test associated with the collective subset returned as significantly different, whilst the local P-values <0.05 (not shown here) for all features.

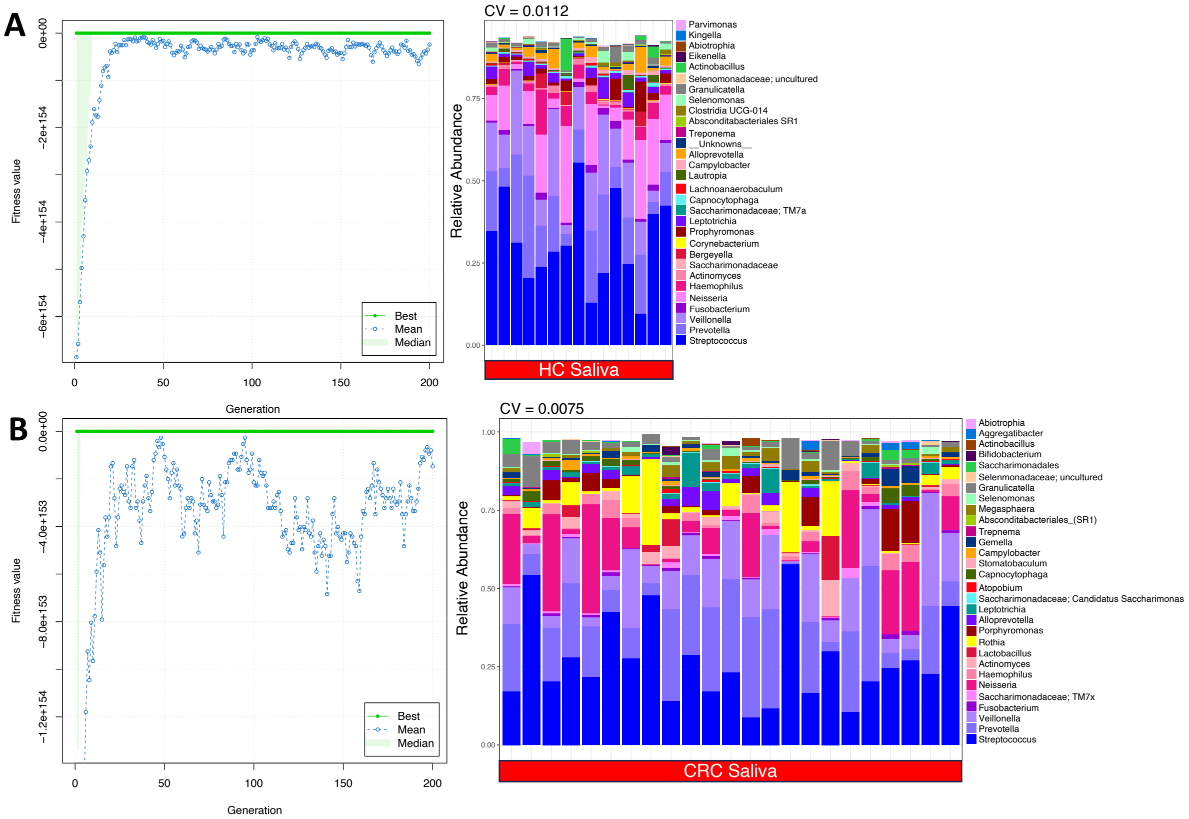

Figure S5: Stable ensemble returned after running EQO algorithm in uniform phenotypic variable mode for (A) HC Saliva, and (B) CRC Saliva samples. The left plots show the fitness value evolution of the genetic algorithm in finding these ensembles highlighting the convergence to a steady state solution whilst the right plots show the relative abundance profiles of these ensembles with *Coefficient of Variation* (CV) values given on the top of the plot. The lower CV value signifies higher stability.

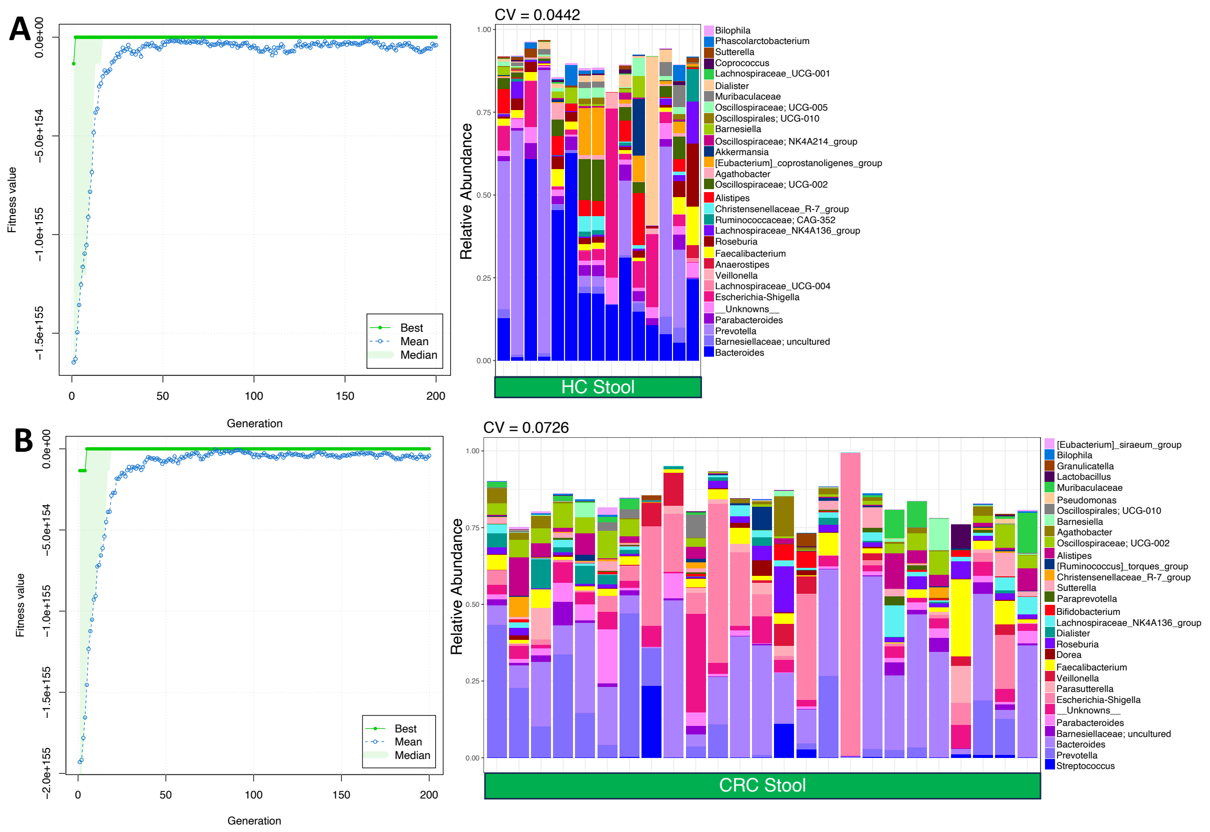

Figure S6: Stable ensemble returned after running EQO algorithm in uniform phenotypic variable mode for (A) HC Stool, and (B) CRC Stool samples. The left plots show the fitness value evolution of the genetic algorithm in finding these ensembles highlighting the convergence to a steady state solution whilst the right plots show the relative abundance profiles of these ensembles with *Coefficient of Variation* (CV) values given on the top of the plot. The lower CV value signifies higher stability.

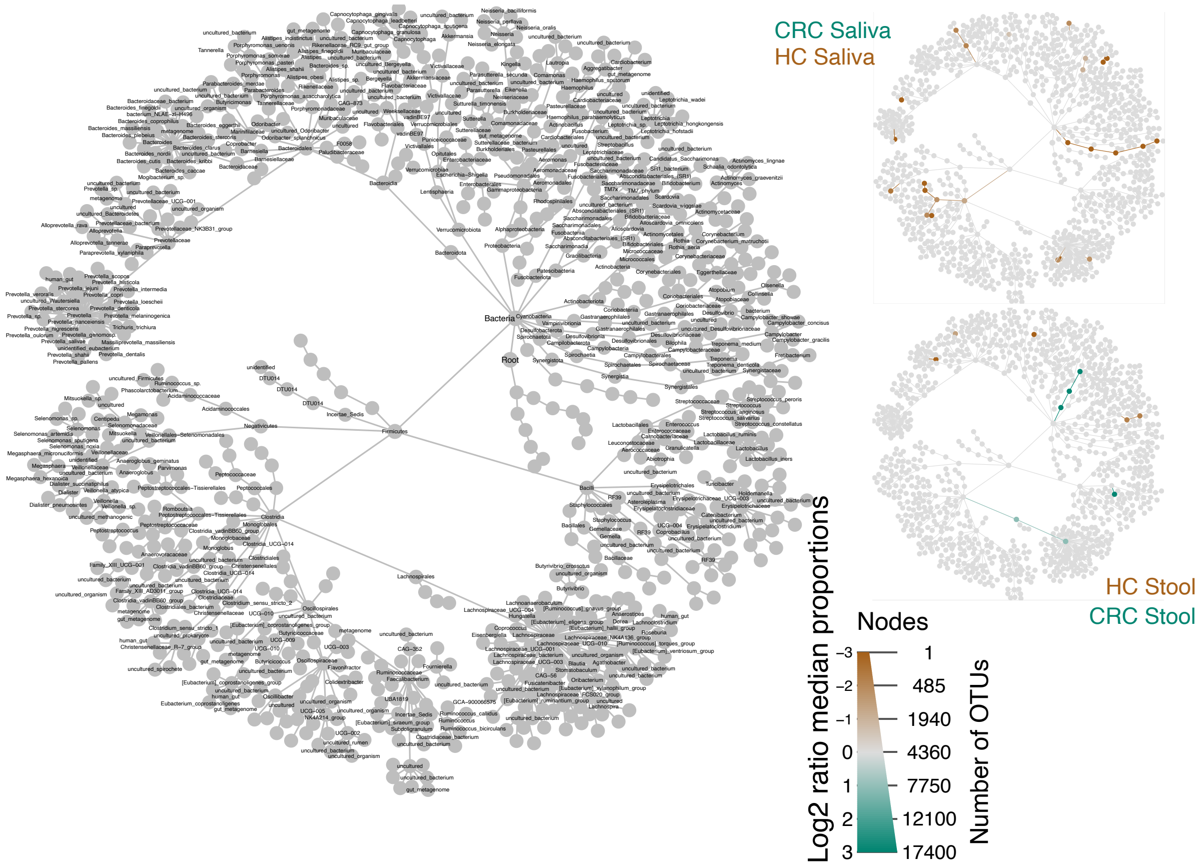

Figure S7: Differential heat tree highlights clades that are differentially where the colour of the branch indicates what it is enriched in. The width of the nodes represents the number of unique OTUs (right of key) at a particular taxonomic level, whilst the colour represents Log2 ratio median proportions of OTUs between the sample. Here grey colour represents the statistically insignificant branches.

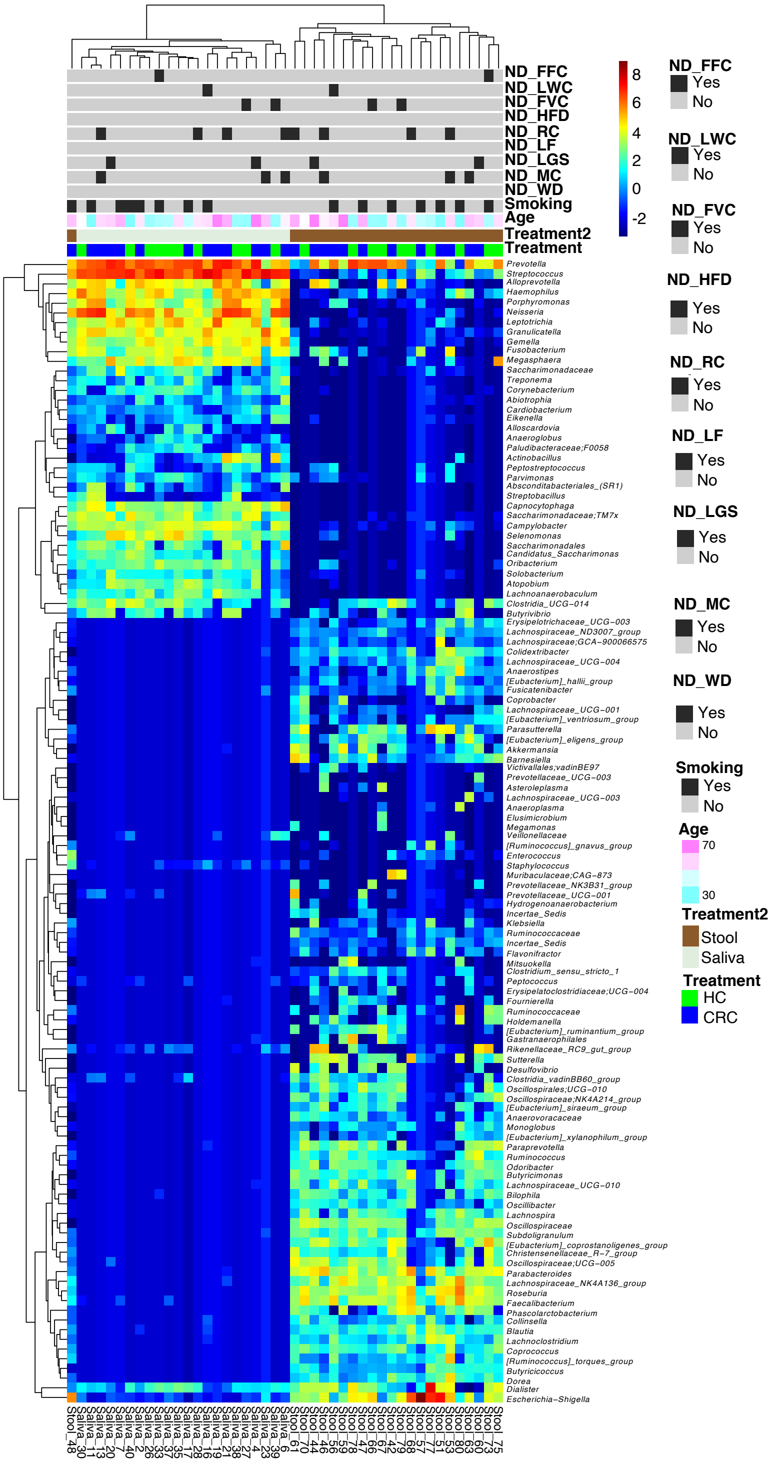

Figure S8: Heatmap of the discriminant genera for MINT algorithm applied for males (as shown in Figure 4), with both rows and columns ordered using hierarchical (average linkage) clustering to identify blocks of genera of interest. Heatmap depicts TSS+CLR normalised abundances, along with metadata drawn on top. Some of the parameters are shown as acronym with details as follow: **ND_FFC** (Nutrition diet-Fast food consumption), **ND_LWC** (Nutrition diet-Less water consumption), **ND_RC** (Nutrition diet-Rice consumption), **ND_LF** (Nutrition diet-Less fruits), **ND_LGS** (Nutrition diet-Low glucose consumption), **ND_MC** (Nutrition diet-Meat consumption), and **ND_WD** (Nutrition diet-western diet).

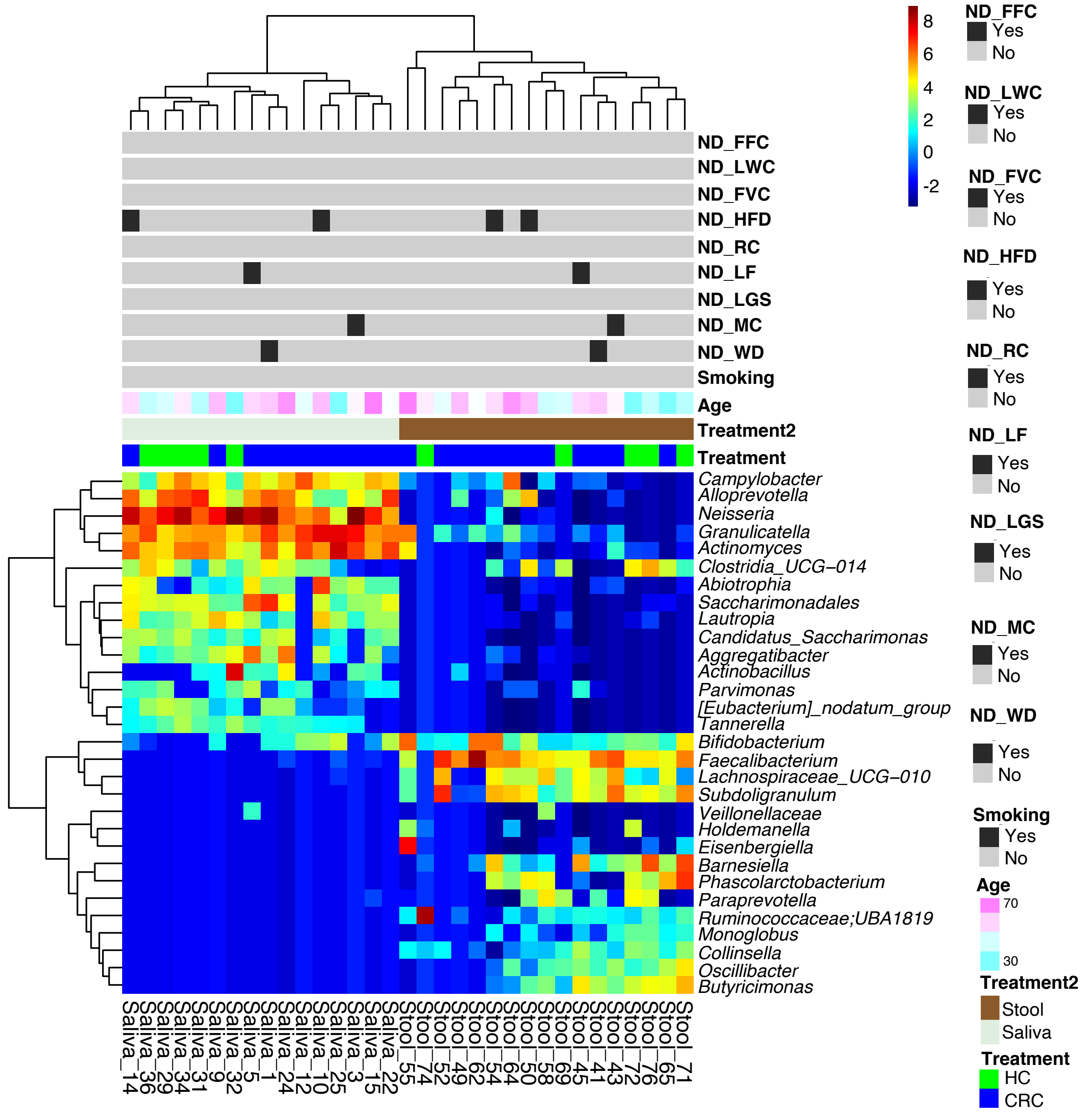

Figure S9: Heatmap of the discriminant genera for MINT algorithm applied for males (as shown in Figure 5), with both rows and columns ordered using hierarchical (average linkage) clustering to identify blocks of genera of interest. Heatmap depicts TSS+CLR normalised abundances, along with metadata drawn on top. Some of the parameters are shown as acronym with details as follow: **ND_FFC** (Nutrition diet-Fast food consumption), **ND_LWC** (Nutrition diet-Less water consumption), **ND_RC** (Nutrition diet-Rice consumption), **ND_LF** (Nutrition diet-Less fruits), **ND_LGS** (Nutrition diet-Low glucose consumption), **ND_MC** (Nutrition diet-Meat consumption), and **ND_WD** (Nutrition diet-western diet).

References

1. Dixon P. VEGAN, a package of R functions for community ecology. Journal of vegetation science. 2003;14(6):927-30.
2. McMurdie PJ, Holmes S. phyloseq: an R package for reproducible interactive analysis and graphics of microbiome census data. PloS one. 2013;8(4):e61217.
3. Zhang Y, Jing G, Chen Y, Li J, Su X. Hierarchical Meta-Storms enables comprehensive and rapid comparison of microbiome functional profiles on a large scale using hierarchical dissimilarity metrics and parallel computing. Bioinformatics Advances. 2021;1(1):vbab003.
4. Love MI, Huber W, Anders S. Moderated estimation of fold change and dispersion for RNA-seq data with DESeq2. Genome biology. 2014;15(12):1-21.
5. Shade A, Stopnisek N. Abundance-occupancy distributions to prioritize plant core microbiome membership. Current opinion in microbiology. 2019;49:50-8.
6. Ijaz UZ, Ameer A, Saleem F, Gul F, Keating C, Javed S. Specialty grand challenge: how can we use integrative approaches to understand microbial community dynamics? : Frontiers Media SA; 2024. p. 1432791.
7. Eng A, Borenstein E. Taxa-function robustness in microbial communities. Microbiome. 2018;6:1-19.
8. Burns AR, Stephens WZ, Stagaman K, Wong S, Rawls JF, Guillemin K, et al. Contribution of neutral processes to the assembly of gut microbial communities in the zebrafish over host development. The ISME journal. 2016;10(3):655-64.
9. Foster ZS, Sharpton TJ, Grünwald NJ. Metacoder: An R package for visualization and manipulation of community taxonomic diversity data. PLoS computational biology. 2017;13(2):e1005404.
10. Rohart F, Eslami A, Matigian N, Bougeard S, Le Cao K-A. MINT: a multivariate integrative method to identify reproducible molecular signatures across independent experiments and platforms. BMC bioinformatics. 2017;18(1):1-13.
11. Gul F, Herrema H, Davids M, Keating C, Nasir A, Ijaz UZ, et al. Gut microbial ecology and exposome of a healthy Pakistani cohort. Gut Pathogens. 2024;16(1):5.
12. Tang Z-Z, Chen G. Robust and powerful differential composition tests for clustered microbiome data. Statistics in Biosciences. 2021;13:200-16.
13. Shan X, Goyal A, Gregor R, Cordero OX. Annotation-free discovery of functional groups in microbial communities. Nature Ecology & Evolution. 2023;7(5):716-24.
